# Perspectives of frail older adults and family caregivers on Urinary Incontinence interventions: A qualitative study

**DOI:** 10.1371/journal.pone.0346758

**Published:** 2026-08-24

**Authors:** Maureen O.’ Callaghan, Dylan Williams, Emma Geraghty, Rachel Hosey, Amanda Clifford, Katie Robinson

**Affiliations:** 1 School of Allied Health, Ageing Research Centre, Health Research Institute, University of Limerick, Ireland; 2 Physiotherapy Department, St. Luke’s Hospital, Kilkenny, Ireland; 3 Health Research Board - Trials Methodology Research Network, University of Limerick, Ireland; Cranfield University, UNITED KINGDOM OF GREAT BRITAIN AND NORTHERN IRELAND

## Abstract

**Background:**

Urinary incontinence (UI) is highly prevalent among older adults living with frailty and is associated with substantial social, emotional, financial, environmental, and caregiver-related burdens. UI is underreported, underassessed and undertreated in this population. Little is known about older adults’ perspectives and experiences of interventions for UI.

**Aim:**

This study aimed to explore the experiences and views of older adults with frailty and family caregivers on conservative, pharmacological and surgical treatments for UI.

**Methods:**

A qualitative descriptive study was conducted using semi-structured interviews with community-dwelling frail older adults experiencing UI and family caregivers. Data were analysed using reflexive thematic analysis.

**Findings:**

Ten participants were recruited (five older adults living with frailty and UI and five family caregivers). Five themes were identified. Theme 1 revealed current self-management strategies for UI and their limitations. Theme 2 describes the prevailing sense of UI treatment nihilism, as participants expressed the belief that UI is an inevitable part of ageing. Older adults with frailty voiced concerns about the potential caregiver burden associated with new treatment approaches, as highlighted in theme 3. Theme 4 indicated a reluctance amongst participants to consider invasive conservative interventions as potential options for UI treatment. In contrast, in theme 5, both older adults with frailty and caregivers displayed a cautious willingness to try new conservative interventions.

**Conclusion:**

Findings indicate support from older adults with frailty and carers for non-invasive conservative interventions for UI. Acceptability was influenced by treatment burden, caregiver involvement, dignity, perceived effectiveness, and beliefs regarding the inevitability of UI in later life.

## Introduction

Ireland is projected to be among the fastest ageing populations in Europe [1], with 26% of the population expected to be aged 65 and over by 2051 [2]. Consequently, the burden of age-associated conditions such as urinary incontinence (UI) is likely to rise significantly.

UI, defined as the involuntary loss of urine [3], affects approximately 25% to 40% of adult women worldwide [4]. Prevalence increases with age and is substantially higher in women than in men, affecting more than 40% of women aged 70 years and older [4]. Rates are particularly high among nursing home residents [5,6]. Despite its high prevalence, UI remains underreported; for example, almost 40% of Irish older adults had not disclosed their UI symptoms to a healthcare professional [7].

UI in older adults is associated with depression, anxiety, decreased physical activity, reduced social engagement, falls, infections, dermatitis, and behavioural disturbances [8–11]. The condition also imposes considerable economic, environmental and societal costs, with the annual burden across the European Union estimated at over €69 billion [12,13]

UI is particularly common among older adults living with frailty, where prevalence is approximately twice that of non-frail peers [14]. Frailty is characterised by reduced physiological reserve and increased vulnerability to adverse health outcomes [15,16]. For this population, conservative, lifestyle and behavioral interventions (such as pelvic floor muscle training, physical exercise, activities of daily living interventions, prompted voiding, attention training, toilet assistance, and lifestyle modifications such as caffeine reduction) are recommended as first line treatment options [17,18] given the potential risks associated with pharmacological and surgical treatments [19]. However, evidence supporting the effectiveness of these interventions specifically in frail populations remains limited [19,20].

Qualitative research has explored the lived experience of UI among older adults and family caregivers, highlighting themes of stigma, symptom concealment, delayed help-seeking, and substantial caregiver burden [21,22]. Collectively, these findings underscore the need for person-centred and caregiver-centred approaches to continence care. However, little is known about how frail older adults and their family caregivers perceive and evaluate available UI interventions.

Perceptions of treatment are important because intervention uptake and adherence are influenced not only by clinical effectiveness but also by perceived acceptability. The Theoretical Framework of Acceptability (TFA) proposes that individuals evaluate healthcare interventions across seven domains: affective attitude, burden, perceived effectiveness, ethicality, intervention coherence, opportunity costs, and self-efficacy [23]. These factors influence whether individuals are willing and able to engage with an intervention and may be particularly relevant for frail older adults managing the complexities of UI alongside other health challenges.

Exploring treatment perceptions may therefore provide important insights to inform the development and implementation of person-centred approaches to continence care. Therefore, the aim of this study was to explore the experiences and perspectives of community-dwelling frail older adults and their family caregivers regarding UI interventions, with a particular focus on their acceptability, feasibility, and perceived barriers.

## Methodology

### Research aims and objectives

A descriptive qualitative design was adopted as it aligned well with the study aim and allows participants to be regarded as experts of their own lived experience with experiential knowledge [24,25]. The study was conducted and is reported in line with the Consolidated Criteria for Reporting Qualitative research (COREQ) guidelines for the reporting of qualitative studies [26]. The COREQ checklist is included in Supplementary Material (S1 File).

### Ethical considerations

Ethical approval was granted by the Health Service Executive South Eastern Area Research Ethics Committee (reference number 23.56). All participants provided written informed consent prior to participation. Participants were provided with an information sheet and an opportunity to ask questions prior to consenting to participate.

### Participant recruitment

A purposive sampling approach was adopted. Community-dwelling men and women aged ≥65 years who were living with frailty and experiencing bothersome UI, along with their family caregivers, were recruited through the Integrated Care Programme for Older Persons (ICPOP) ambulatory care clinic in the Midlands region of Ireland (Carlow and Kilkenny). Access to these multidisciplinary specialist services (Community Specialist Teams for Older Persons, CST-OP) requires that older adults have been identified as frail—defined as a score of 4–7 on the Rockwood Clinical Frailty Scale (CFS) [15]—and be aged 65 years or older.

Posters displayed in the ICPOP site facilitated self-referral, additionally older adults meeting the following inclusion criteria were invited to participate via a gatekeeper (Nurse or team member working at the ICPOP site): aged ≥ 65 years old, living with frailty or vulnerable to frailty, defined as scoring between 4 and 7 on the Rockwood CFS, experiencing self -reported or clinician diagnosed UI > 6 months, able to hear and follow instructions and with the capacity to provide informed consent to participate in an interview. Older adults meeting any of the following criteria were excluded from the study: participants with a history of urogynaecological/pelvic/prostate cancers, participants with an indwelling urinary catheter or participants with mental health/psychiatric disorders or neurological /neurodegenerative disorders. Family carers were required to meet the following inclusion criteria: self-reported family carer providing daily care support to an older person with frailty and UI, aged 18 years or over, conversational English language skills and the capacity to provide informed consent to participate in a one-to-one interview. A recent cohort study indicates that CST-OP attendees are typically older adults with complex health needs, high levels of frailty and multimorbidity, and an elevated risk of adverse outcomes including functional decline, hospitalisation, nursing home admission, and mortality [27].

Recruitment was conducted in two phases. Phase 1 took place between July and November 2023. Eligible patients and family caregivers identified by the gatekeeper who expressed interest in participating were contacted by the lead researcher (MO’C). After reviewing the study information and having the opportunity to ask questions, a date and time for the interview were scheduled by MO’C with those interested in participating. Recruitment was slower than anticipated and was paused after Phase 1 while the lead researcher (MO’C) completed submission requirements for a MSc by Research degree. A second phase of recruitment occurred between November and December 2024 and followed the same recruitment procedures. However, interviews were conducted by MO’C or EG and DW following recruitment by MO’C. No service changes occurred between the two phases of data collection. In all cases, written consent forms were posted to participants in advance of the interview. Interviews were scheduled at least seven days after the initial telephone contact to allow participants sufficient time to consider the study information and to withdraw if they so wished. Interviewers had no prior relationship with interviewees.

### Data collection and management

Semi-structured, one-to-one interviews were conducted either face to face in participants’ own homes or via telephone and were audio recorded. All face-to-face interviews were conducted by the lead researcher (MO’C) in participants’ homes. Telephone interviews were conducted jointly by EG and DW with both researchers present for the duration of the interview. The same interview guide and probing approach were used across face-to-face and telephone interviews. Telephone interviews were offered to reduce participant burden and accommodate participant preferences. Given the sensitive nature of UI, only the interviewer(s) and the participant were present during interviews. Field notes were recorded immediately following each interview by the interviewer(s) and were subsequently transcribed in full. No repeat interviews were conducted. Interviews were audio recorded.

This interview guide was developed by MO’C and KR, informed by the findings of a systematic literature review of conservative non-pharmacological interventions for UI in frail older adults aged ≥65 years [20] and explores the lived experience of urinary incontinence among older people with frailty and their family caregivers, beginning with the nature, onset, and daily impact of symptoms. It examines how urinary incontinence affects physical functioning, social participation, relationships, and emotional wellbeing. The guide also investigates help-seeking behaviours, treatment history, and day-to-day management strategies, including pharmacological, surgical, and conservative approaches. Participants are asked to reflect on their experiences with healthcare services and treatment outcomes. Finally, it explores views on future engagement with evidence-based conservative interventions (prospective acceptability as described by the TFA), including factors influencing participation, adherence, and perceived barriers. Probing questions were used to deepen exploration and allow unstructured discussion, facilitating rich data collection. Such flexibility is particularly important when exploring sensitive topics, as it allows participants to control the extent of disclosure [28]. Interviewers were sensitive to the topic and emphasised that participants could decline to answer any questions. Interviewees knew the profession of interviewers. All participants completed a brief demographic questionnaire following the interview.

Prior to data collection, all interviewers were familiarised with the study aims, relevant literature, and the semi-structured interview guide. In advance of data collection MO’C engaged in discussions with KR regarding the distinctions between clinical and qualitative research interviewing and the use of probing techniques. Before conducting interviews, EG and DW reviewed the interview materials and relevant literature and participated in a dedicated preparation session, which included rehearsal of the interview schedule, discussion of challenges associated with interviewing older adults about urinary incontinence, practice using probes, and consideration of reflexive interviewing approaches. Researchers also engaged in reflective discussions following interviews to refine the interview process. The semi-structured interview guide was used across all interviews to ensure consistency in the topics explored; however, flexibility was maintained to allow participants to shape the discussion and to accommodate individual needs, including fatigue, fluctuating health status, and differing levels of comfort discussing UI.

### Reflexivity

All interviewers were novice qualitative researchers; however, EG (woman) and DW (man), MSc Occupational Therapy pre-registration students, had prior experience conducting clinical interviews and working with older adults through practice education placements, while MO’C (woman) is a qualified physiotherapist (BSc & MSc) with extensive clinical experience working with frail older adults and individuals living with UI. Analysis of all data was supervised by KR, an occupational therapist (BSc, MSc, PhD) and experienced qualitative researcher with expertise in older adult rehabilitation.

### Sample adequacy

Sample adequacy was guided by the concept of information power, which suggests that studies with a narrow aim and highly specific, information-rich participants may require fewer participants [29]. Recruitment ceased when the research team judged that sufficient information power had been achieved, based on the focused study aim, the specificity of the sample, and the richness of the data generated regarding experiences of urinary incontinence interventions among frail older adults and their family caregivers. Notably, as described in the results section participants represented a highly specific and information-rich sample of older adults with moderate to severe frailty and complex health needs, and family caregivers closely involved in continence management and treatment decision-making.

### Data analysis

Recorded interviews and field notes were transcribed verbatim by MO’C, DW or EG and checked for accuracy against the recordings before being imported into NVivo Version 14 (QSR 2019) for analysis. Data were analysed by MOC, DW, EG and KR using a six stage, reflexive approach to thematic analysis as described by Braun and Clarke [30–33]. The emphasis on reflexivity encouraged continuous reflection on the researchers’ role and potential influence across all stages of the research process. The first stage was getting acquainted with the data, through transcription of the audio recordings, followed by reading and rereading the data which allowed the researchers to engage in in-depth immersion in the data. Throughout this procedure, initial ideas and concepts were noted to inform the following stage. Data were then all coded inductively at a descriptive, semantic level, focusing on participants’ explicit accounts and the manifest content of the transcripts rather than imposing a framework or the research teams’ ideas and pre-conceptions on the data. All data segments considered relevant to the study aim were coded. Code labels and definitions were refined iteratively throughout the process, with codes being merged, split, and revised as understanding of the dataset developed. Analytic decisions were supported through regular research team meetings involving discussion, reflection, and critical examination of interpretations. The third phase involved searching for themes by identifying patterns across the coded data. Related and overlapping codes were grouped together to develop preliminary themes that captured shared meanings and patterns of response across participants. In the fourth phase, candidate themes were reviewed and refined to ensure they accurately reflected patterns within the coded data and the dataset as a whole. During the fifth phase, themes were defined and named, with clear boundaries established between themes. The sixth and final phase involved producing the report, integrating data extracts and analytic interpretation to present a coherent account of the findings in relation to the research question. MOC coded the initial six interviews, EG and DW coded the final 4 interviews. All steps were supported by a second experienced qualitative researcher (KR) who read the transcripts, coded three transcripts, was centrally involved in the identification and refinement of themes and provided critical feedback throughout the thematic analysis process. Discrepancies were resolved through discussion. The lead researchers (MO’C, DW & EG) throughout the process of data collection and analysis, maintained a thorough record of their reflexive consideration of their values, beliefs, and previous experiences to ensure reflexive consideration of their impact on the research process. Regular discussions were scheduled between the research team throughout the analysis process.

## Results

### Participant demographic

Eight older adults and their respective family caregivers were recruited; however, three dyads subsequently withdrew due to changes in health or social circumstances. Consequently, five older adults and five family caregivers completed the study. Eight participants were interviewed at home and two via telephone.

All recruited older adult participants were classified as having moderate to severe frailty according to the Rockwood Clinical Frailty Scale. Participants’ sociodemographic characteristics are presented in Table 1. Pseudonyms were assigned to all participants to ensure de-identification. Older adult participants ranged in age from 74 to 93 years, with a mean age of 83.2 years (SD 6.98). Four family caregivers were adult children and one was a spousal caregiver. Interview durations ranged from 31 to 42 minutes. Transcripts were not returned to participants for member checking, and participants did not provide feedback on the study findings.

**Table 1:** Participant demographic details.

Pseudonym	Age	Gender	Frequency of UI	Caregiver type	Interviewed by
Helen Older Adult	82	Woman	Daily	NA	M’OC
Carmel Older Adult	81	Woman	Daily	NA	EG/DW
Tim Older Adult	93	Man	>3 times/week	NA	MO’C
Margaret Older Adult	86	Woman	>3 times/day	NA	MO’C
Mike Older Adult	74	Man	> 3 times/week to 1/day	NA	MO’C
Tom Caregiver	85	Man	NA	Spouse	EG & DW
Louise Caregiver	56	Woman	NA	Adult daughter	MO’C
Liz Caregiver	60	Woman	NA	Adult daughter	MO’C
Shauna Caregiver	63	Woman	NA	Adult daughter	MO’C
Bill Caregiver	47	Man	NA	Adult son	MO’C

### Overview of themes

Five overarching themes were identified from the interviews: (1) inadequate current urinary incontinence (UI) management strategies, (2) UI treatment nihilism, (3) caregiver capacity and burden influencing engagement with UI interventions, (4) reluctance to try invasive interventions, medication or surgery, and (5) openness to conservative UI interventions.

### Inadequate current UI management strategies

Participants described a range of strategies or treatments used to manage UI, including use of absorbent incontinence pads/ products, anticipatory toileting, use of toileting aids such as urine bottles, modification of clothes, restriction of fluid intake and or caffeine, and avoidance of constipation. Most participants described reluctant acceptability of these due to associated negative consequences as illustrated in the following quote related to fluid restriction and resultant dehydration.

*Tomorrow night he will be going down to the village. There will be a GAA* [Gaelic Athletic Association] *celebration and he’ll find it hard there. He won’t drink a whole lot… it’s in the back of his mind the whole time not to be drinking too much. By not drinking his water then he tends to get dehydrated sometimes* (Bill, caregiver)*.*

Participants’ experiences of managing UI while maintaining participation in everyday activities varied considerably. Some older adults described successful strategies such as ensuring access to toilet facilities and using continence products, allowing them to continue participating in valued activities with relatively little disruption.

*No, the urinary leaking doesn’t stop me doing anything. But I’d have to know there was a toilet close by. If I had to go to a new place, I would hate it, yeah. First thing, I would enquire about is where the toilets were, and I always have to wear a pad.* (Margaret, Older Adult).

However, many more described restricting or avoiding activities due to UI and fear of leakage in public.

*I don’t go to as many GAA matches as I used to because of the urine problem and the walking* (Mike, Older Adult).

Similarly, caregivers described avoiding social events due to concerns about managing UI in public settings:

*Now there was a family wedding there back a couple of weeks ago, and, no, we didn’t go because that that can be messy (Tom, Caregiver).*

For participants who had been referred to a Public Health Nurse (PHN), support was generally viewed positively and PHNs were regarded as an important source of assistance with UI management. However, experiences of the continence products supplied through PHN services were more varied. While participants valued access to absorbent products, some caregivers felt that the products provided did not adequately meet the individual continence needs or preferences of the older adult. As one caregiver explained:

*They’re not comfortable or easy to use… they’re a bit cumbersome. It’s kind of one size fits all. He likes the pull up ones, but they’re not sent out to him* (Bill, caregiver)

Similar concerns were raised by another caregiver, who described finding alternative products because the standard continence pads supplied were uncomfortable for her mother:

*She doesn’t like the incontinence ones* [pads] *because they’re quite bulky, so we go for the heavy period flow pads because they work better for her. She finds them more comfortable, but we just have to change them more regularly* (Louise, caregiver).

Two older adults had previously tried medication for UI, but neither reported a positive experience. One found the medication ineffective, while the other discontinued treatment due to intolerable side effects: *“The side effects were awful and that I was completely dried up, my mouth and severe constipation, and it was just horrible. So the remedy was worse than the actual problem” (Carmel, Older Adult).*

One older adult reported limited benefit from previous UI-specific surgery and pelvic floor exercises.

### UI treatment nihilism

This theme captures a pervasive sense of treatment nihilism among frail older adults and their caregivers, characterised by resignation about living with UI and pessimism regarding UI treatment options. UI was consistently described as an inevitable and irreversible consequence of ageing and associated physical decline, rather than a treatable condition. More broadly, participants described worsening UI over time, which they often attributed to declining mobility and increasing frailty.

Nihilism was also identified in reports of passive acceptance of symptoms and reduced urgency to seek further support. As one older adult stated:

*Listen, there’s nothing much they can do anyway. Sure, I’m old in the first place. There are no tablets or anything that I know of that could make this stop* (Mike, Older Adult).

Although some noted embarrassment most participants did discuss UI with their GP

*It was embarrassing to say it to the doctor even. You know that I have this problem* (Carmel, Older Adult).

However, treatment nihilism was also reflected in accounts of clinical encounters as noted by one participant:

*Maybe I did tell the GP and was told there wasn’t anything or any treatment they could offer me. Everything was because of my age, you know?* (Margaret, Older Adult).

Similarly, other accounts noted no management plan or referral pathway for UI. However, an important tension emerged within this theme. Although participants often expressed pessimism regarding cure, they did not necessarily reject intervention. Three older adults and a further three caregivers noted that even modest improvements in symptoms would be valued

*If I got a small bit better, I’d say I’d be happy with that* (Margaret, Older Adult).

Differences between older adults and caregivers were also evident. Older adults tended to internalise age-related explanations for UI and express resignation. In contrast, caregivers were more likely to describe frustration at what they perceived as missed opportunities for earlier intervention when their family member was less frail and when treatment could have been more effective.

*She was actually never offered treatment. I suppose because she’s old now. Yeah, like maybe it would have worked at the very start, earlier when she was up and pretty independent. (Shauna, Caregiver).*

### Caregiver capacity and burden affecting engagement with UI interventions

Caregiver burden was described as a key factor shaping intervention acceptability and feasibility. Older adult participants reported being highly aware of the demands placed on caregivers and expressed gratitude for the support they received, including transport to appointments, help managing health conditions and UI, and assistance with daily activities. Caregivers themselves described the emotional, practical, occupational, and financial impact of supporting older adults living with frailty and UI.

*Well, it’s not just the urinary leakage. It’s a combination of everything, really. Everything that’s happening, where he’s going. It’s having a huge impact on me. I’m thinking that I’m just going to have to manage my funds as I’m thinking about cutting back my hours in work* (Liz, caregiver)

Older adults also expressed concern that additional UI treatments might increase caregiver burden. Distance, competing responsibilities, and limited availability were seen as barriers to engaging in further interventions. As one older adult reflected, requesting help with exercises or completing a bladder diary would take additional time from family members who already had significant commitments (Margaret, Older Adult).

Despite these challenges, caregivers consistently expressed willingness to support interventions that might improve symptoms.

*We would just try anything really, any exercises to help with the bladder* (Shauna, Caregiver).

This created a notable tension within the data. While older adults often worried about burdening family members, caregivers generally expressed willingness to help. However, this willingness was conditional on interventions being feasible within existing caregiving responsibilities. For example:

*Prompted voiding might work too, although that would be putting pressure on us to remember to prompt him, especially during our working day* (Bill, Caregiver).

The home setting was reported by older adults and carers as the preferred location for treatment by the older adults, due to comfort and to minimise caregiver burden. There was mixed acceptability of clinic and group settings for interventions.

### Reluctance to try invasive conservative interventions, medication or surgery

This theme describes the reluctance of all older adults and most caregivers to engage with invasive conservative interventions, medication, or surgery. All participants considered surgical interventions unacceptable due to the associated risks such as age-related risk factors with concerns about potential complications and past experiences with unsuccessful surgeries.

*He’s too old for surgery. He wouldn’t go for surgery* (Liz, caregiver).

Medication was approached with greater nuance. Participants expressed caution regarding side effects but remained open to medication if recommended by a trusted healthcare professional and if benefits outweighed risks.

*Well, if they could do anything to help me, including tablets, I wouldn’t say no if they offered to do something for me* (Margaret, Older Adult).

Similarly, views regarding invasive conservative interventions were mixed rather than uniformly negative. While some participants rejected approaches involving electrical stimulation with vaginal or anal probes or biofeedback with a probe or HCP’s digit due to concerns regarding privacy, dignity, or discomfort:

*I don’t, wouldn’t feel very comfortable at the thought of that. I wouldn’t be happy at all doing that biofeedback. I wouldn’t. I’m a very private person* (Tim, Older Adult).

One caregiver (adult daughter) expressed willingness to support invasive interventions (e.g., vaginal probe) but felt the older adult would be uncomfortable and unlikely to consent.

*I don’t know if he’d like that now. It wouldn’t bother me, and I wouldn’t be in the least bit squeamish…I don’t know whether he’d be comfortable with that treatment, you’d have to ask him that now, but it wouldn’t bother me at all (Liz, Caregiver).*

One additional caregiver was open to such interventions, while three were not. Concerns regarding preservation of dignity and potential emotional discomfort for the older adult were shared across all caregivers. Some caregivers questioned whether mobility limitations would make some invasive interventions difficult to complete at home, regardless of potential effectiveness.

### Willingness to Try Conservative UI Interventions

This theme reflects the general acceptability of conservative UI interventions among frail older adults and their caregivers. All participants (n = 10) expressed cautious willingness to try non-pharmacological approaches, including prompted voiding, pelvic floor muscle training, bladder diaries, bladder training with urge suppression techniques, lifestyle modifications, and newer non-invasive options such as transcutaneous tibial nerve stimulation (TTNS), particularly when these were perceived as low risk and capable of providing even modest symptom improvement. As one participant explained:

*I think there wouldn’t be any harm in that. If it’s for the better, I would do prompted voiding* (Tim, Older Adult).

Similarly, caregivers were willing to support participation in these interventions. Participants were also receptive to interventions targeting factors beyond the urinary tract itself, including mobility, strength, environmental modifications, and assistive devices. Such interventions were often viewed as particularly relevant because participants linked worsening UI to broader declines in mobility and physical functioning.

Despite broad acceptability, participants highlighted potential challenges to implementation. Cognitive changes, limited supervision, travel requirements for clinic-based interventions, competing health priorities, and caregiver availability were identified as barriers to conservative interventions. One caregiver described prior difficulties maintaining adherence to exercises due to forgetfulness and boredom, while another noted the potential challenges of supporting lifestyle changes such as diet changes for UI when the older adult lived alone.

*Yeah, but look, he lives on his own. There’s no one here to keep on eye on what he’s eating and drinking during the whole day*” (Bill, Caregiver).

In addition, one older adult expressed concern about their physical capacity to engage in certain conservative interventions, despite being interested in trying them.

## Discussion

This qualitative study explores the perspectives of community-dwelling older adults with frailty (≥65 years) and their caregivers on treatments for UI. Participants described inadequate current care and expressed treatment nihilism. While there was cautious openness to conservative interventions, concerns about caregiver burden and feasibility were prominent, alongside reluctance toward invasive, pharmacological, or surgical treatments.

Treatment nihilism is an important factor influencing care seeking and health provider behaviour. Participants frequently viewed UI as an inevitable part of ageing and reported it received limited clinical attention with missed opportunities for earlier intervention. These findings are consistent with previous research linking poor UI management to therapeutic nihilism, low disclosure rates, and gaps in healthcare professional knowledge, highlighting the need for improved awareness, early identification, and evidence-based management of UI [34–37].

This study also provides important insights into the acceptability of UI treatments in frail older adults. Participants generally favoured less invasive approaches and expressed reluctance towards surgery, pharmacological treatments, and invasive conservative interventions. Surgical interventions were generally considered unacceptable due to perceived risks, consistent with evidence of poorer surgical outcomes in frail populations [38–40]. Participants were similarly cautious regarding pharmacological treatments, reflecting concerns about adverse effects and the limited efficacy and tolerability of many UI medications in older adults [36,41–43]. Invasive conservative interventions, such as probe-based electrical stimulation and biofeedback, were also viewed less favourably due to concerns about dignity, privacy, and discomfort. These findings align with previous research demonstrating a preference among older adults for interventions that are less invasive, easy to use, and non-embarrassing [44].

Participants were open to engaging with non-invasive conservative, non-pharmacological interventions as first-line treatment, consistent with international guideline recommendations [17,18,36]. Interventions such as bladder training, pelvic floor muscle exercises, dietary modification, and other behavioural strategies, which are perceived as low-risk and practical approaches [21,45]. However, evidence for these interventions in community-dwelling frail older adults remains limited, with most research conducted in nursing home settings [20]. Given this evidence gap, and the known importance of acceptability for successful implementation [23], our findings support the need for future effectiveness studies and implementation efforts tailored to the specific needs and preferences of this group. Such research should address barriers to intervention uptake and adherence, including travel, multimorbidity, cognitive impairment, caregiver dependence, and limited support for those living alone.

Caregiver burden was a major factor influencing treatment acceptability. Older adults were often reluctant to pursue interventions that might increase caregiver workload, while caregivers described significant emotional, social, and practical impacts associated with continence care, consistent with previous research [21,46–49]. Importantly, despite these impacts, caregivers were willing to support conservative UI interventions. Consequently, future intervention studies should carefully consider caregiver capacity and include appropriate support mechanisms [50]. Previous research suggests caregivers value practical training and individualised support to manage continence effectively [21].

Importantly, participants emphasised that treatment success did not necessarily require cure. Instead, even modest reductions in symptom frequency or severity were viewed as potentially meaningful. This aligns with recommendations that outcomes for UI interventions in frail populations should extend beyond symptom reduction to include quality of life, social participation, caregiver outcomes, and cost-effectiveness [21,36,51]. These findings highlight the importance of selecting outcomes in effectiveness studies that reflect what matters most to frail older adults and their caregivers when evaluating UI interventions.

### Strengths and limitations

This study provides one of the first qualitative explorations of the acceptability of UI treatments among frail older adults and their family caregivers [22,52]. While previous qualitative research has examined the lived experience of incontinence, caregiver burden, and continence management in populations such as people living with dementia, little attention has been paid to how frail older adults and caregivers evaluate specific treatment options [22]. Our findings extend the existing literature by identifying key factors influencing intervention acceptability, including treatment burden, caregiver involvement, dignity, feasibility, and treatment nihilism.

A key strength is the inclusion of both older adults and caregivers, recognising that perspectives on UI management may differ across stakeholders. Interviews were conducted in participants’ homes in four cases, which supported comfort when discussing a sensitive topic and by telephone in one case which reduced travel burden for this population.

However, several limitations should be noted. Recruitment of frail older adults is challenging due to fluctuating health status, comorbidities, and competing healthcare demands, which likely contributed to the low participation rate despite a total recruitment window of six months. In addition, all older adult participants were living with moderate to severe frailty, and therefore findings may not reflect the perspectives of those across the wider frailty spectrum. As participants were recruited through the CST-OP service, the findings may not fully reflect the perspectives of frail older adults who are less engaged with, or have more limited access to, healthcare services. Despite these limitations, consistent patterns emerged across interviews, suggesting the themes identified capture important aspects of UI management in this population.

Future research should replicate this study with a larger and more diverse sample of frail older adults with UI and their caregivers to confirm and extend these findings. A stratified approach to sampling in future studies may be useful to ensure the view of particularly vulnerable older adults are represented. Recent studies have indicated that women of lower socioeconomic status are at increased risk of UI [53]. Recruitment strategies should account for the challenges of engaging frail populations, including fluctuating health status, competing medical appointments, accessibility issues, and reliance on caregiver support.

### Practice recommendations

These findings support the use of conservative, non-pharmacological interventions as first-line management for UI in frail older adults, consistent with current clinical guidelines. Healthcare providers should actively screen for UI, promote earlier identification and referral, and address treatment nihilism that may delay care. Interventions should be tailored to the individual circumstances of older adults and their caregivers, recognising the potential for caregiver burden and consider opportunities for home-based care delivery.

## Supporting information

S1 File Supplementary Material COREQ Checklist. (DOCX)
