# Supplementary material for: Perspectives of frail older adults and family caregivers on Urinary Incontinence interventions: A qualitative study
Source: PLoS One. 2026 Aug 24;21(8):e0346758. doi: 10.1371/journal.pone.0346758 (PMC13502618; doi:10.1371/journal.pone.0346758)
Supplement: Supplementary file 2 [file pone.0346758.s003.docx]

**Response to Reviewers**

PONE-D-26-14259 **Perspectives of Frail Older Adults and Family Caregivers on Urinary Incontinence Interventions: A Qualitative Study**

We would like to thank the reviewers for their thoughtful and constructive comments, as well as for recognising the strengths and potential contribution of this study. We greatly appreciate the time and expertise invested in reviewing our manuscript.

We have carefully considered all comments and suggestions and have revised the manuscript accordingly. We believe these revisions have strengthened the clarity, rigour, and overall quality of the paper. A detailed, point-by-point response to each comment is provided below, with corresponding changes highlighted in the revised manuscript.

We hope that the revisions satisfactorily address the reviewers’ concerns and we thank the reviewers again for their valuable feedback.

| **Editor comment** | **Author response** | **Page #** |
| --- | --- | --- |
| a) If there are ethical or legal restrictions on sharing a de-identified data set, please explain them in detail (e.g., data contain potentially identifying or sensitive patient information, data are owned by a third-party organization, etc.) and who has imposed them (e.g., a Research Ethics Committee or Institutional Review Board, etc.). Please also provide contact information for a data access committee, ethics committee, or other institutional body to which data requests may be sent. | We thank the reviewer for this suggestion. While we support the principles of data sharing where appropriate, we are unable to make the full dataset publicly available. The study involved a small sample recruited from a single site, which is identifiable through the named ethics committee. Given the sensitive nature of the topic and the inclusion of detailed personal information relating to family circumstances, living situations, health conditions, and care arrangements, there is a risk that participants could be identifiable even following de-identification of transcripts. In addition, ethical approval for public sharing of the full dataset was not obtained, and data sharing was not included in the participant information materials or consent process. Queries regarding the ethical approval of the study can be directed to:  Research Ethics Committee Office Old School of Nursing University Hospital Waterford Dunmore Road Waterford, Ireland  REC Coordinator: Pauline Prendergast Tel: +353 (0)51 842 026 Email: [SERegionalResearchEthics@hse.ie](mailto:SERegionalResearchEthics@hse.ie) |  |
| Please amend either the title on the online submission form (via Edit Submission) or the title in the manuscript so that they are identical. | They are identical, namely; Perspectives of Frail Older Adults and Family Caregivers on Urinary Incontinence Interventions: A Qualitative Study |  |
| **Reviewer comment** | **Author response** | **Page #** |
| Tighten up the background section. There is considerable epidemiological evidence, and it takes quite a bit of text to arrive at the study aims. | The Introduction has been revised and condensed. Epidemiological and background information has been streamlined, the detailed discussion of frailty has been reduced, and the rationale for the study and associated knowledge gap are now introduced earlier. | Page 3-5. |
| Background: Is there a conceptual framework (e.g. a health behavior theory, or something of the like) that could be introduced to this section? Such an addition would more prominently set up *why* treatment perceptions matter, and not just that they are understudied. | Thank you for this suggestion. We agree that the rationale for exploring treatment perceptions could be strengthened through reference to a conceptual framework. We have therefore introduced the Theoretical Framework of Acceptability (TFA) in the Introduction. | Pg. 8 |
| Methods: Please clarify the rationale for the chosen sample size - was thematic saturation was achieved? Alternatively, what was the rationale for analytic sufficiency given the small, purposively selected sample?  Reviewer 2 :  1. The study includes ten participants (five older adults and five caregivers), which is acceptable in qualitative research; however, the manuscript does not sufficiently justify how this sample size was determined.  • How was the final sample size decided? • Was data saturation considered? • What indicators were used to determine that sufficient depth and breadth of data had been achieved? | Thank you to both reviewers for highlighting the need for more clarity on sample size.  We agree that clarification regarding sample size and analytic sufficiency is warranted. This study employed purposive sampling to recruit information-rich participants with direct experience of urinary incontinence and intervention use within the context of frailty. Consistent with the concept of information power, sample adequacy was determined by the specificity of the study aim, the relevance and richness of participant experiences, and the depth of the interview data. Participants represented an information-rich group, comprising community-dwelling older adults with moderate to severe frailty and complex health needs many of whom had lived with UI for decades, alongside family caregivers closely involved in continence management and treatment decision-making. We have added a section to the Methods section to clarify this rationale. | End of page 9 / start of page 10 |
| Please provide additional description of interviewer training and standardization, particularly given the use of multiple interviewers with varying levels of experience and mixed interview modalities (in-person and telephone). Were any steps taken to ensure consistency?  3. Data collection occurred in two phases, with variation in interviewer configuration (single vs paired interviewers) and mode (face-to-face vs telephone).  • How were these differences managed to ensure consistency across interviews? • What steps were taken to minimise variability introduced by different interviewers or formats? • Could these differences have influenced the depth or nature of the data collected? | Thank you to both reviewers for highlighting this.  We agree that further detail regarding interviewer preparation and consistency is warranted. Prior to data collection, all interviewers were familiarised with the study aims, relevant literature, and the semi-structured interview guide. The lead interviewer (MO'C), an experienced clinician with expertise in urinary incontinence and older adult care, developed the interview schedule in collaboration with the research team and under the supervision of KR. During this process, discussions focused on the distinctions between clinical and qualitative research interviewing, the use of probing techniques, and strategies for exploring a sensitive topic while supporting participant comfort and disclosure. Reflective discussions were undertaken following early interviews to refine the interview approach.  Before conducting interviews, EG and DW reviewed the study materials, interview guide, and relevant literature and participated in a dedicated preparation session with KR. In preparation for data collection, they also engaged with the urinary incontinence and qualitative methods literature over a five-month period as part of their MSc research training. The preparation session included rehearsal of the interview schedule, discussion of the challenges associated with interviewing older adults about urinary incontinence, practice using probes, and consideration of reflexive interviewing approaches. Following interviews, researchers engaged in reflective discussions regarding interview conduct, participant responses, and opportunities to enhance subsequent data collection.  While a common interview guide was used to ensure all key topics were addressed across interviews, full standardisation was not sought given the semi-structured nature of the interviews. Flexibility was maintained to allow participants to shape the discussion and to accommodate individual needs, including fatigue, fluctuating health status, and differing levels of comfort discussing urinary incontinence.  With regard to interview modality, the same semi-structured interview guide was used for both face-to-face and telephone interviews. Telephone interviews were offered to reduce participant burden and to respond to participant preference. The interview approach, topics explored, and use of probes were consistent across modalities.  Additional text has been added to the Methods section to clarify these procedures. | Page 8 and 9 |
| Methods: The description of the analytic approach is particularly thin in comparison to the rest of the methods. Please describe in more depth, including how coding decisions were made, how discrepancies were resolved, and the role of the senior qualitative researcher. | The process of RTA has been detailed more explicitly. | Page 10 and 11 |
| Data analysis 1. While reflexive thematic analysis is described, the manuscript would benefit from greater transparency regarding the analytic process.  • How were coding decisions made collaboratively across researchers? • Were there disagreements during coding or theme development, and how were these resolved? • How did the research team actively reflect on and manage their own assumptions during analysis? | The process of RTA has been detailed more explicitly. | Page 10 and 11. |
| 2. Participants were recruited via a gatekeeper within an ICPOP service, which may introduce selection bias.  • How might this recruitment approach have influenced the characteristics of the sample? • Could participants be more engaged with healthcare services than the broader population of frail older adults? • How might this affect the transferability of the findings? | Thank you for highlighting this consideration. Participants were recruited through the ICPOP Community Specialist Team for Older Persons (CST-OP) service and via study advertisements displayed within the clinic and through gatekeepers. We acknowledge that individuals engaged with CST-OP services may be more connected to healthcare services than some frail older adults in the wider community, which may have influenced the perspectives captured. To provide greater context regarding the population from which participants were recruited and to support readers in assessing transferability, we have added a description of CST-OP attendees based on a recent prospective cohort study. This demonstrates that CST-OP users are an older, clinically complex population with high levels of frailty, multimorbidity, falls risk, and vulnerability to adverse outcomes. We have also acknowledged in the limitations section that the recruitment approach may have resulted in the inclusion of participants who were more engaged with healthcare services than some community-dwelling frail older adults, which should be considered when interpreting the transferability of the findings. | Methods page 7 line 124-127 and Limitations page 23 |
| Results: 1. These data are so important for the field, but could benefit from deepened analysis that moves beyond description to more interpretive insights that *explain* phenomenon of interest. I would personally like to see a comparison of perspective between older adults and caregivers.  Results  2. Theme boundaries could be refined to reduce conceptual overlap and improve clarity - find places to reduce repetition.  Results 3. I would like to see stronger intentionality with data excerpts - to not only demonstrate that a theme is present, but also to show how it varies across participants and contexts, including areas of tension or nuance. This revision would strengthen both the credibility and interpretive depth of the findings. | We thank the reviewer for these constructive comments. In response, we revisited the original codes and re-engaged with the interview data to further deepen the analysis. The Results section has been substantially revised to move beyond description and provide greater interpretive insight into the factors influencing the acceptability of UI interventions among frail older adults and their caregivers.  To address Comment 1, we strengthened the analytic narrative throughout the findings, focusing on explaining how and why particular interventions were viewed as acceptable or unacceptable. Greater attention has been paid to comparing and contrasting the perspectives of older adults and family caregivers.  To address Comment 2, we reviewed and refined theme boundaries to reduce conceptual overlap and improve clarity. Repetitive content was removed or consolidated, and themes were revised to ensure that each captured a distinct aspect of intervention acceptability. Some content not clearly connected to the research question has been deleted. In particular, clearer distinctions were made between treatment nihilism, caregiver burden, and willingness to engage with future interventions.  To address Comment 3, we revisited the selection and use of participant quotations. We also strengthened the interpretive commentary surrounding quotations to demonstrate not only the presence of themes but also the complexity of participants' experiences. | Page 13 onwards |
| Discussion: This section would benefit from a stronger shift from description to explanation and integration. While the authors appropriately link findings to existing literature and clinical guidelines, the section remains largely confirmatory. What are the mechanisms underlying key findings - for example, *why* does treatment nihilism persist? How does caregiver burden shape intervention feasibility and decision-making? | In response, we substantially revised the Discussion to provide greater interpretation of the findings and explore potential mechanisms underlying key themes. Caregiver burden and the differing views of older adults and carers are explored on page 22. | Page 20 onwards |
| The implications section could also be strengthened by offering more specific, actionable guidance for intervention design and implementation, particularly around caregiver-inclusive models and feasibility in real-world settings. | Throughout the discussion implications for future intervention research are more detailed and specific. |  |
| Discussion and Conclusion  1. Some conclusions, particularly regarding the “urgent need” for improved healthcare provider assessment and treatment, appear stronger than what the qualitative data alone can support.  • Are these claims directly grounded in the data presented? • Could the conclusions be more cautiously framed to reflect the exploratory nature of the study? | References to ‘urgent need’ have been removed and more moderate claims made reflective of the study findings. | Page 23/4. |
| 2. The manuscript states that this is the first qualitative study focusing specifically on urinary incontinence treatment perspectives in a frail older adult population.  • Can this claim be fully substantiated? • Would it be more appropriate to frame this as “to our knowledge” or “one of the first”? • Could the authors clarify what specific aspect of the study is novel (e.g. focus on frailty, treatment perspectives, or inclusion of caregivers)? | We agree that our original wording may have overstated the novelty of the study. In response, we have revised the manuscript to describe this work as “one of the first qualitative explorations” rather than the first study of its kind.  We have also clarified the specific contribution of the study. While previous qualitative research has explored the lived experience of urinary incontinence, caregiver burden, and continence management, including among populations such as people living with dementia, there remains very limited research examining how frail older adults and their family caregivers evaluate the acceptability of specific urinary incontinence treatment options. The novelty of the present study lies in its focus on (1) frail community-dwelling older adults, (2) perceptions of treatment acceptability rather than experiences of living with incontinence alone, and (3) the inclusion of both older adult and caregiver perspectives. | Pg. 23 |
