# Supplementary material for: Perspectives of frail older adults and family caregivers on Urinary Incontinence interventions: A qualitative study
Source: PLoS One. 2026 Aug 24;21(8):e0346758. doi: 10.1371/journal.pone.0346758 (PMC13502618; doi:10.1371/journal.pone.0346758)
Supplement: Peer review history

# Peer review: Decision Letter 0

**DOI:** 10.1371/journal.pone.0346758.r001

**Authors:** Alison Parker

5 May 2026

Dear Dr. Robinson,

Please submit your revised manuscript by Jun 19 2026 11:59PM. If you will need more time than this to complete your revisions, please reply to this message or contact the journal office at plosone@plos.org. When you're ready to submit your revision, log on to https://www.editorialmanager.com/pone/ and select the 'Submissions Needing Revision' folder to locate your manuscript file.

- A letter that responds to each point raised by the academic editor and reviewer(s). You should upload this letter as a separate file labeled 'Response to Reviewers'.
- A marked-up copy of your manuscript that highlights changes made to the original version. You should upload this as a separate file labeled 'Revised Manuscript with Track Changes'.
- An unmarked version of your revised paper without tracked changes. You should upload this as a separate file labeled 'Manuscript'.

If you would like to make changes to your financial disclosure, please include your updated statement in your cover letter. Guidelines for resubmitting your figure files are available below the reviewer comments at the end of this letter.

If applicable, we recommend that you deposit your laboratory protocols in protocols.io to enhance the reproducibility of your results. Protocols.io assigns your protocol its own identifier (DOI) so that it can be cited independently in the future. For instructions see: https://journals.plos.org/plosone/s/submission-guidelines#loc-laboratory-protocols. Additionally, PLOS ONE offers an option for publishing peer-reviewed Lab Protocol articles, which describe protocols hosted on protocols.io. Read more information on sharing protocols at https://plos.org/protocols?utm_medium=editorial-email&utm_source=authorletters&utm_campaign=protocols.

As the corresponding author, your ORCID iD is verified in the submission system and will appear in the published article. PLOS supports the use of ORCID, and we encourage all coauthors to register for an ORCID iD and use it as well. Please encourage your coauthors to verify their ORCID iD within the submission system before final acceptance, as unverified ORCID iDs will not appear in the published article. *Only*  the individual author can complete the verification step; PLOS staff *cannot*  verify ORCID iDs on behalf of authors.

We look forward to receiving your revised manuscript.

Kind regards,

Alison Parker

Academic Editor

PLOS One

Journal Requirements:

When submitting your revision, we need you to address these additional requirements.

1. Please ensure that your manuscript meets PLOS ONE's style requirements, including those for file naming. The PLOS ONE style templates can be found at

https://journals.plos.org/plosone/s/file?id=wjVg/PLOSOne_formatting_sample_main_body.pdf and

https://journals.plos.org/plosone/s/file?id=ba62/PLOSOne_formatting_sample_title_authors_affiliations.pdf

2. We note that you have indicated that there are restrictions to data sharing for this study. For studies involving human research participant data or other sensitive data, we encourage authors to share de-identified or anonymized data. However, when data cannot be publicly shared for ethical reasons, we allow authors to make their data sets available upon request. For information on unacceptable data access restrictions, please see http://journals.plos.org/plosone/s/data-availability#loc-unacceptable-data-access-restrictions.

Before we proceed with your manuscript, please address the following prompts:

a) If there are ethical or legal restrictions on sharing a de-identified data set, please explain them in detail (e.g., data contain potentially identifying or sensitive patient information, data are owned by a third-party organization, etc.) and who has imposed them (e.g., a Research Ethics Committee or Institutional Review Board, etc.). Please also provide contact information for a data access committee, ethics committee, or other institutional body to which data requests may be sent.

b) If there are no restrictions, please upload the minimal anonymized data set necessary to replicate your study findings to a stable, public repository and provide us with the relevant URLs, DOIs, or accession numbers. Please see http://www.bmj.com/content/340/bmj.c181.long for guidelines on how to de-identify and prepare clinical data for publication. For a list of recommended repositories, please see https://journals.plos.org/plosone/s/recommended-repositories. You also have the option of uploading the data as Supporting Information files, but we would recommend depositing data directly to a data repository if possible.

Please update your Data Availability statement in the submission form accordingly.

3.Please amend either the title on the online submission form (via Edit Submission) or the title in the manuscript so that they are identical.

4. If the reviewer comments include a recommendation to cite specific previously published works, please review and evaluate these publications to determine whether they are relevant and should be cited. There is no requirement to cite these works unless the editor has indicated otherwise.

[Note: HTML markup is below. Please do not edit.]

Reviewers' comments:

Reviewer's Responses to Questions

**Comments to the Author**

1. Is the manuscript technically sound, and do the data support the conclusions?

Reviewer #1: Partly

Reviewer #2: Yes

**********

2. Has the statistical analysis been performed appropriately and rigorously?

Reviewer #1: N/A

Reviewer #2: Yes

**********

3. Have the authors made all data underlying the findings in their manuscript fully available?

The PLOS Data policy

Reviewer #1: No

Reviewer #2: No

**********

4. Is the manuscript presented in an intelligible fashion and written in standard English?

Reviewer #1: Yes

Reviewer #2: Yes

**********

Reviewer #1: This manuscript addresses an important and underexplored area: treatment perspectives on urinary incontinence (UI) among frail older adults and caregivers. The focus on acceptability and perceived feasibility of interventions is a clear strength and aligns with contemporary patient-centered and implementation-oriented research priorities.

Strengths include the inclusion of both older adults and caregivers, capturing the dyadic nature of care and highlighting caregiver burden as a key factor in treatment decision-making. The qualitative design is well described, is appropriate for the study questions and yields clinically actionable insights, particularly the preference for non-invasive interventions and the role of treatment nihilism. Overall, the findings have practical relevance for improving patient-centered and caregiver-informed continence care.

There are some areas on which I suggest revision:

Background section:

1.Tighten up the background section. There is considerable epidemiological evidence, and it takes quite a bit of text to arrive at the study aims.

2. Is there a conceptual framework (e.g. a health behavior theory, or something of the like) that could be introduced to this section? Such an addition would more prominently set up *why* treatment perceptions matter, and not just that they are understudied.

Methods:

1. Please clarify the rationale for the chosen sample size - was thematic saturation was achieved? Alternatively, what was the rationale for analytic sufficiency given the small, purposively selected sample?

2. Please provide additional description of interviewer training and standardization, particularly given the use of multiple interviewers with varying levels of experience and mixed interview modalities (in-person and telephone). Were any steps taken to ensure consistency?

3. The description of the analytic approach is particularly thin in comparison to the rest of the methods. Please describe in more depth, including how coding decisions were made, how discrepancies were resolved, and the role of the senior qualitative researcher.

Results:

1. These data are so important for the field, but could benefit from deepened analysis that moves beyond description to more interpretive insights that *explain* phenomenon of interest. I would personally like to see a comparison of perspective between older adults and caregivers.

2. Theme boundaries could be refined to reduce conceptual overlap and improve clarity - find places to reduce repetition.

3. I would like to see stronger intentionality with data excerpts - to not only demonstrate that a theme is present, but also to show how it varies across participants and contexts, including areas of tension or nuance. This revision would strengthen both the credibility and interpretive depth of the findings.

Discussion:

1. This section would benefit from a stronger shift from description to explanation and integration. While the authors appropriately link findings to existing literature and clinical guidelines, the section remains largely confirmatory. What are the mechanisms underlying key findings - for example, *why* does treatment nihilism persist? How does caregiver burden shape intervention feasibility and decision-making?

2. The implications section could also be strengthened by offering more specific, actionable guidance for intervention design and implementation, particularly around caregiver-inclusive models and feasibility in real-world settings.

Reviewer #2: Overall, the manuscript addresses an important and underexplored topic and makes a valuable contribution to the field. It has clear potential for publication, and I hope the following comments will be helpful in improving the manuscript.

Comments

Methods

1. The study includes ten participants (five older adults and five caregivers), which is acceptable in qualitative research; however, the manuscript does not sufficiently justify how this sample size was determined.

• How was the final sample size decided?

• Was data saturation considered?

• What indicators were used to determine that sufficient depth and breadth of data had been achieved?

2. Participants were recruited via a gatekeeper within an ICPOP service, which may introduce selection bias.

• How might this recruitment approach have influenced the characteristics of the sample?

• Could participants be more engaged with healthcare services than the broader population of frail older adults?

• How might this affect the transferability of the findings?

3. Data collection occurred in two phases, with variation in interviewer configuration (single vs paired interviewers) and mode (face-to-face vs telephone).

• How were these differences managed to ensure consistency across interviews?

• What steps were taken to minimise variability introduced by different interviewers or formats?

• Could these differences have influenced the depth or nature of the data collected?

Data analysis 1. While reflexive thematic analysis is described, the manuscript would benefit from greater transparency regarding the analytic process.

• How were coding decisions made collaboratively across researchers?

• Were there disagreements during coding or theme development, and how were these resolved?

• How did the research team actively reflect on and manage their own assumptions during analysis?

Discussion and Conclusion

1. Some conclusions, particularly regarding the “urgent need” for improved healthcare provider assessment and treatment, appear stronger than what the qualitative data alone can support.

• Are these claims directly grounded in the data presented?

• Could the conclusions be more cautiously framed to reflect the exploratory nature of the study?

2. The manuscript states that this is the first qualitative study focusing specifically on urinary incontinence treatment perspectives in a frail older adult population.

• Can this claim be fully substantiated?

• Would it be more appropriate to frame this as “to our knowledge” or “one of the first”?

• Could the authors clarify what specific aspect of the study is novel (e.g. focus on frailty, treatment perspectives, or inclusion of caregivers)?

**********

what does this mean?). If published, this will include your full peer review and any attached files.

If you choose “no”, your identity will remain anonymous but your review may still be made public.

**Do you want your identity to be public for this peer review?**  For information about this choice, including consent withdrawal, please see our Privacy Policy

Reviewer #1: No

Reviewer #2: No

**********

[NOTE: If reviewer comments were submitted as an attachment file, they will be attached to this email and accessible via the submission site. Please log into your account, locate the manuscript record, and check for the action link "View Attachments". If this link does not appear, there are no attachment files.]

To ensure your figures meet our technical requirements, please review our figure guidelines: https://journals.plos.org/plosone/s/figures

You may also use PLOS’s free figure tool, NAAS, to help you prepare publication quality figures: https://journals.plos.org/plosone/s/figures#loc-tools-for-figure-preparation.

NAAS will assess whether your figures meet our technical requirements by comparing each figure against our figure specifications.

# Author comment: Author response to Decision Letter 1

**DOI:** 10.1371/journal.pone.0346758.r002

29 Jun 2026

Dear Editor and Reviewers, thank you for your review of our manuscript. We include a detailed document with a point by point response to all issues you raised.

Attachment Submitted filename: Response to Reviewers.docx

# Editor report: Decision Letter 1

**DOI:** 10.1371/journal.pone.0346758.r003

**Authors:** Alison Parker

22 Jul 2026

Re: Perspectives of Frail Older Adults and Family Caregivers on Urinary Incontinence Interventions: A Qualitative Study

PONE-D-26-14259R1

Dear Dr. Robinson,

We’re pleased to inform you that your manuscript has been judged scientifically suitable for publication and will be formally accepted for publication once it meets all outstanding technical requirements.

Within one week, you’ll receive an e-mail detailing the required amendments. When these have been addressed, you’ll receive a formal acceptance letter and your manuscript will be scheduled for publication.

An invoice will be generated when your article is formally accepted. Please note, if your institution has a publishing partnership with PLOS and your article meets the relevant criteria, all or part of your publication costs will be covered. Please make sure your user information is up-to-date by logging into Editorial Manager at Editorial Manager® and clicking the ‘Update My Information' link at the top of the page. For questions related to billing, please contact billing support.

If your institution or institutions have a press office, please notify them about your upcoming paper to help maximize its impact. If they’ll be preparing press materials, please inform our press team as soon as possible -- no later than 48 hours after receiving the formal acceptance. Your manuscript will remain under strict press embargo until 2 pm Eastern Time on the date of publication. For more information, please contact onepress@plos.org.

Kind regards,

Alison Parker

Academic Editor

PLOS One

Additional Editor Comments (optional):

Reviewers' comments:
